# Fougera’s Cod Liver Oil

**Published:** 1868-08-15

**Authors:** 


### FOUGERA'S COD-LIVER OIL.

DURING the past autumn and winter, a trial was made at the out-door department of Bellevue Hospital of the Iodinized Cod-Liver Oil, prepared by Mr. Fougera, of William street, with the view to ascertain whether or not this oil possessed any advantages over the ordinary uncombined cod-liver oils.

Before giving the results it is fair to say that no other kind of practice presents so few facilities for forming a decided opinion of the merits and efficacy of any medicine, as that of a dispensary. In a hospital the physician has the assurance that his directions in regard to the administration of the medicine will be faithfully carried out, and has moreover generally an opportunity of observing the result. In private practice also he has this latter advantage, though not always the former. In a dispensary practice he has neither. The medicine may or may not be properly administered. If the patient recovers, he generally thinks it unnecessary to come back to report his cure: if he thinks he is not improving, he will probably change to some other dispensary, and the case is lost. An opinion must be formed from the few cases that continue under treatment throughout the case, or by observing whether the patient had improved from visit to visit, though the cure was yet incomplete. The advantages claimed for Mr. Fougera's cod-liver oil, are that by reason of the addition of iodine, bromine, and phosphorus, it is more efficacious, and at the same time the stomach need not be disordered by an excessive amount of oil administered. This oil was given to about eighty patients, about thirty of whom were children, the remainder belonging chiefly to the department of chest diseases. Owing to the difficulties above mentioned, no statistical account of the result can be given; but the opinion of the physicians using it is nearly unanimous to this effect: that the oil is of decided medicinal value; that compared with ordinary cod-liver oil, it appears to take effect more rapidly; and that it obviates the very common necessity of adding extemporaneously to the oil, medicines containing iodine or iron, particularly the syrup of iodide of iron. In private practice, where the price of the article used is not of much importance, it would be worth while to give this preparation a trial.—*Medical Gazette.*

---

*Bromide of Ammonium in Delirium Tremens.*—Dr. J. B. Fares, of Romeo, Mich., says:

"I have recently had a case of delirium tremens which yielded promptly to the use of *Bromide of Ammonium*, in ten grain doses, every two hours. Quiet sleep secured after second dose, and calm sleep within twenty-four of the commencing dose. Patient had had two previous attacks within the past two years, neither of which yielded within seven or eight days. I have seen the *Bromide of Potassium* recommended in the difficulty, but believe the *Ammonium* (therapeutically considered) far superior to the *Potassium*, on account of the mild stimulant afforded by the *Ammonia*."

